# Genome-Wide Identification and Low-Temperature Expression Analysis of bHLH Genes in *Prunus mume*

**DOI:** 10.3389/fgene.2021.762135

**Published:** 2021-10-01

**Authors:** Aiqin Ding, Anqi Ding, Ping Li, Jia Wang, Tangren Cheng, Fei Bao, Qixiang Zhang

**Affiliations:** Beijing Key Laboratory of Ornamental Plants Germplasm Innovation and Molecular Breeding, Beijing Laboratory of Urban and Rural Ecological Environment, Key Laboratory of Genetics and Breeding in Forest Trees and Ornamental Plants of Ministry of Education, Engineering Research Center of Landscape Environment of Ministry of Education, National Engineering Research Center for Floriculture, Beijing Forestry University, Beijing, China

**Keywords:** *Prunus mume*, basic helix-loop-helix gene family, genome-wide analysis, expression pattern, low temperature stress 3

## Abstract

*Prunus mume* is an illustrious ornamental woody plant with colorful flowers, delicate fragrances, and graceful tree forms. Low temperature limits its geographical distribution. The basic helix-loop-helix (bHLH) proteins exist in most eukaryotes as a transcription factor superfamily, which play a crucial role in metabolism, physiology, development, and response to various stresses of higher organisms. However, the characteristics of the bHLH gene family and low-temperature response remain unknown in *P. mume*. In the present study, we distinguished 95 *PmbHLH* genes in the *P. mume* whole-genome and analyzed their features. *PmbHLHs* were divided into 23 subfamilies and one orphan by phylogenetic analysis. Similar gene structures and conserved motifs appeared in the same subfamily. These genes were situated in eight chromosomes and scaffolds. Gene duplication events performed a close relationship to *P. mume*, *P. persica,* and *P. avium*. Tandem duplications probably promoted the expansion of *PmbHLHs*. According to predicted binding activities, the PmbHLHs were defined as the Non-DNA-binding proteins and DNA-binding proteins. Furthermore, *PmbHLHs* exhibited tissue-specific and low-temperature induced expression patterns. By analyzing transcriptome data, 10 *PmbHLHs* which are responsive to low-temperature stress were selected. The qRT-PCR results showed that the ten *PmbHLH* genes could respond to low-temperature stress at different degrees. There were differences in multiple variations among different varieties. This study provides a basis to research the evolution and low-temperature tolerance of *PmbHLHs*, and might enhance breeding programs of *P. mume* by improving low-temperature tolerance.

## Introduction

Plants are subject to various unsuitable environmental stresses when they grow in a natural environment. Low-temperature stress is a severe natural disaster, which divides into chilling stress and freezing stress. Chilling stress mainly affects the process of photosynthesis and respiratory metabolism of plants, resulting in the disorder of plant cell function to make them grow abnormally and even causing growth stagnation (Pearce, 1988). Freezing stress freezes plant cells, then causes mechanical damage to the plant cell membrane and eventually might cause plant death (Mccully et al., 2004; Knight et al., 2009). To adapt and resist low-temperature stress, plants have evolved a set of complex and fine regulation mechanisms. Transcription factors are vital in plant signal regulatory networks. When plants suffer from low-temperature stress, transcription factors can activate low-temperature responsive genes by binding *cis*-acting elements on gene promoters. Thus, they regulate signal transduction pathways to improve low-temperature tolerance in plants.

The basic helix–loop–helix (bHLH) proteins, which belong to superfamily transcription factors are widely spread in plants, animals, and fungi (Ledent and Vervoort, 2001). The bHLH superfamily contains two highly conserved domains: the basic region and helix-loop-helix (HLH) region (Atchley et al., 1999). The basic region is composed of 15–20 amino acids and is located at the N-terminal of the bHLH domain, which can recognize and bind DNA (Atchley and Fitch, 1997). In the plant bHLH domain, 50% of the basic region contains a highly conserved His5-Glu9-Arg13 sequence, which can bind to E-box (5′-CANNTG-3′) element. This is necessary for bHLH to bind to DNA (Pires and Dolan, 2010). In the C-terminus of the bHLH domain, the HLH region is composed of about 40 amino acids. This region is characterized by two α-helices connected by a loop with variable length (Murre et al., 1989). The HLH domain can promote interaction between proteins to form homodimers or heterodimers and interact with E-box elements in the genes promoter region (Massari and Murre, 2000; Huq and Quail, 2002). Therefore, the biological functions of most bHLH transcription factors involve forming dimers.

The bHLH transcription factors are related to plant growth and development (Groszmann et al., 2008; Ding et al., 2009), floral organ formation (Buti et al., 2020), secondary metabolism (Nemesio-Gorriz et al., 2017; Wang et al., 2019), and stress resistance (Chinnusamy, 2003; Zhou et al., 2009; Seo et al., 2011; Gao et al., 2020). Under low-temperature stress, many studies have proved that bHLHs are involved in regulation. For example, *ICE1* (INDUCER OF CBF EXPRESSION 1), which belongs to the bHLH transcription factor family, could activate *CBF3* and *COR* genes in response to low temperature in *Arabidopsis thaliana*. Meanwhile, other bHLH transcription factors could also regulate cold tolerance in plants. In rice seedlings, cold stress specifically induced *OsbHLH1* gene expression (Wang et al., 2003). Apple *MdCIbHLH1* played a role in cold tolerance in a CBF dependent manner (Feng et al., 2012).

*Prunus mume* Sieb. et Zucc, a crucial woody plant with excellent ornamental characteristics for various colors, delicate fragrances, ample flower shapes, and abundant tree forms has been widely used for plant landscaping. *P. mume* originated in *the* Yangtze River Basin and Southwest China. The cultivar was distributed in Northern China and East Asia, with domestication taking place over a long time (Zhang et al., 2018). However, low temperature is still a limiting factor for the northward distribution of *P. mume*. Therefore, it is essential to enhance cold resistance to expand distribution. The bHLH transcription factors have been proved to be involved in resisting low-temperature stress. Nevertheless, the identification of bHLH genes has still not been conducted in *P. mume*. In the present study, we identified 95 *PmbHLH* genes and performed a comprehensive bioinformatics analysis based on *P. mume* genome-wide. We analyzed gene identification, phylogenetic tree, DNA binding activity, gene structure, conserved motifs, protein interaction, chromosomal distribution, synteny analysis, and tissue-specific expression. Combining RNA sequencing data and qRT-PCR analysis, the expression patterns of *PmbHLHs* were estimated under low-temperature stress. Overall, our results could form the foundation for researching the biological function of *PmbHLHs* and enrich the low-temperature resistance gene bank in woody plants.

## Material and Methods

### Genome-Wide Identification of *PmbHLHs*

We applied the genome project (http://prunusmumegenome.bjfu.edu.cn) to download the whole genome data of *P. mume* (Zhang et al., 2012). The sequences of *A. thaliana* bHLHs (AtbHLHs) were obtained from Pires and Dolan (Pires and Dolan, 2010). The Pfam database (http://pfam.xfam.org, PF00010) was used to obtain the Hidden Markov Model (HMM) profile of the HLH domain and was searched bHLH proteins of *P. mume* with HMMER3 software (http://hmmer.janelia.org) (Sun et al., 2015). To ensure credibility, the E-value cut-off was set at 10^–5^. SMART online software (http://smart.embl-heidelberg.de/) (Schultz et al., 1998) and NCBI CD-search (http://www.ncbi.nlm.nih.gov/Structure/cdd/wrpsb.cgi) (Marchler-Bauer et al., 2015) were used to confirm bHLH domains in presumptive PmbHLH proteins of *P. mume*.

The WoLF PSORT program (https://wolfpsort.hgc.jp/) was applied to predict the subcellular localization of *PmbHLHs* (Horton et al., 2007). The CDS length, molecular weights (MWs), theoretical isoelectric points (pI), amino-acid sequences (aa), the total number of positively charged residues (Arg + Lys), total number of negatively charged residues (Asp + Glu), grand averages of hydropathicity (GRAVYs), and instability index and aliphatic index of all predicted *PmbHLHs* were calculated using ExPASy (https://web.expasy.org/protparam/) (Artimo et al., 2012).

### Phylogenetic Analysis and Multiple Alignment

RAxML version eight software with maximum likelihood (ML) method was used to construct phylogenetic trees (Stamatakis, 2014). The optimal JTT (Jones-Taylor-Thornton) model of amino acid substitution was applied to construct ML phylogenetic trees. The calculated relationships of the phylogenetic tree were supported by performing 1,000 iterations of bootstrap test and visualized by iTOL (https://itol.embl.de/) (Letunic and Bork, 2006). Alignments of PmbHLHs domains were conducted by Muscle (https://www.ebi.ac.uk/Tools/msa/muscle/) (Edgar, 2004).

### Gene Structure and Conserved Domain

TBtools (Chen et al., 2020) and NCBI Batch CD-Search (https://www.ncbi.nlm.nih.gov/) (Marchler-Bauer et al., 2015) were applied to analyze and visualize gene structure and conserved domains. We applied the online MEME program to analyze motif structures of PmbHLH proteins (30 motifs were set as the maximum number), others using default parameters (Bailey et al., 2009). Jalview software (Clamp et al., 2004) and Weblogo3 (http://weblogo.berkeley.edu/logo.cgi) were used to visualize and analyze conserved domains.

### Chromosomal Distribution, Gene Duplication, and Synteny

We used the GDR database (https://www.rosaceae.org/) to retrieve genomes of *Prunus avium* and *Prunus persica* (Verde et al., 2013; Shirasawa et al., 2017). The *P. mume* genome database provided chromosomal distribution information of *bHLH* genes. Chromosomal location map, as well as duplication events of *bHLH* genes and mutation rates of Ka (nonsynonymous) versus Ks (synonymous) were predicted through TBtools (Chen et al., 2020). Syntenic relationship of *bHLH* genes was analyzed by MCScanX (Multiple Collinearity Scan toolkit) in *P. mume*, *P. avium,* and *P. persica* (Wang et al., 2012). We used the R circlize package to visualize the relationship between three varieties (Zhuo et al., 2018). The *PmbHLH* genes divergence time (T) was calculated through the equation: T = dS/(2λ × 106) Mya, where λ = 1.5 × 10^−8^ s for dicots (Lynch and Conery, 2000).

### Gene Expression Analysis

The transcriptome data of *PmbHLHs* in five tissues (bud, fruit, leaf, stem, and root) were downloaded from the NCBI Sequence Read Archive (accession number: SRP014885). Heat map of five tissues and low-temperature stress were drawn by TBtools (Chen et al., 2020) and iTOL (https://itol.embl.de/) based on FPKM values (Letunic and Bork, 2006).

### Plant Materials and Treatments

Annual grafted seedlings of true *mume* ‘Beijing Yudie’ and apricot mei ‘Danfenghou’ were used for low-temperature treatment. These plant materials were exposed to the same soil humidity (60–70%), air humidity (65–70%), illumination time (12 h/12 h), and light intensity (150 μmol•m^−2^•s^−1^). Stems of *P. mume* were treated for chilling treatment (4°C for 8 h and 5 days) and freezing treatment (−5°C for 1 h).

To further research the regulation process of *PmbHLHs* under low-temperature, we carried out detailed multi-stage chilling treatment and freezing treatment.

1) Chilling treatment: in September, we took annual branches of ‘Beijing Yudie” and put branches in a 4°C low-temperature incubator and undertook sampling at 0/2/4/8/16/24/48/72 h. Stem segments without bud points were used as sampling materials.
2) Freezing treatment: in November, when the lowest ambient temperature reaches 5°C, we took branches of 'Beijing Yudie', then put them in a refrigerator at 4°C. The second day, they were put in a low-temperature incubator with step-by-step cooling (1 h/°C, keeping it from 0°C to −10°C). Stem segments were sampled at 0/2/4/6/8/10/24/48 h. The above samples were stored at −80°C for RNA extraction. There were three biological replicates.

### RNA Extraction and qRT-PCR Analysis

Total RNA of low-temperature treatments was isolated by RNA Extraction Kit (Takara, Beijing, China) based on references. First-strand cDNA synthesis was reversed with DNase-treated RNA (1 µg) through PrimeScript^TM^RT Reagent Kit with gDNA Eraser (Takara, Dalian, China). The template used cDNA (2 µL) in a 10 µL qRT-PCR by TB Green Ⅱ Premix Ex Taq (Takara, Dalian, China). We applied the 2^ΔΔCt^’ method to calculate relative expression levels and internal control used protein phosphatase 2A (PP2A)gene of *P. mume* (Wang et al., 2014). Supplementary Table S10 showed ten selected genes and specific primers. Each qRT-PCR was repeated at least three times.

### Promoter *Cis*-Acting Elements and Protein Interaction Analysis

Promoter *cis*-acting regulatory elements were analyzed by PlantCARE (http://bioinformatics.psb.ugent.be/webtools/plantcare/html/) (Lescot et al., 2002). The AraNet V2 tool (Lee et al., 2015) was used to construct the protein interaction network based on homologous proteins of PmbHLHs in *Arabidopsis*. The protein interaction network was visualized by Cytoscape (Shannon, 2003) and STRING software (http://string-db.org/).

## Results

### Identification of *PmbHLHs* Genes in *P. Mume*

A total of 95 non-redundant *PmbHLHs* were discovered based on HMMER software from *P. mume* genome. According to their location position, these genes were named *PmbHLH01* to *PmbHLH95*. The length of PmbHLH proteins ranged between 91 (PmbHLH36) and 700 (PmbHLH45) amino acids and most of these genes (74%) had lengths of 200–400 aa. The presumptive isoelectric points (pI) ranged from 4.57 (PmbHLH14) to 10.1 (PmbHLH43). About molecular weight values, the smallest was 10.27 kDa (PmbHLH67) and the largest was 78.26 kDa (PmbHLH45). The instability index varied from 36.78 to 92.34, while only one (PmbHLH66) was considered a stable protein. Predicted GRAVY values were all negative, representing all genes that possessed hydrophilic characteristics. The aliphatic index, (Asp + Glu) value and (Arg + Lys) value of PmbHLHs showed diversity features. Analysis of the gene structure of 95 PmbHLH proteins showed that coding genes of most PmbHLH proteins (92.63%) have introns. Subcellular localization of PmbHLH proteins were mostly located in the nucleus (Supplementary Table S1).

### Phylogenetic Analysis, Multiple Sequence Alignment, and DNA-Binding Activity

**FIGURE 1:**
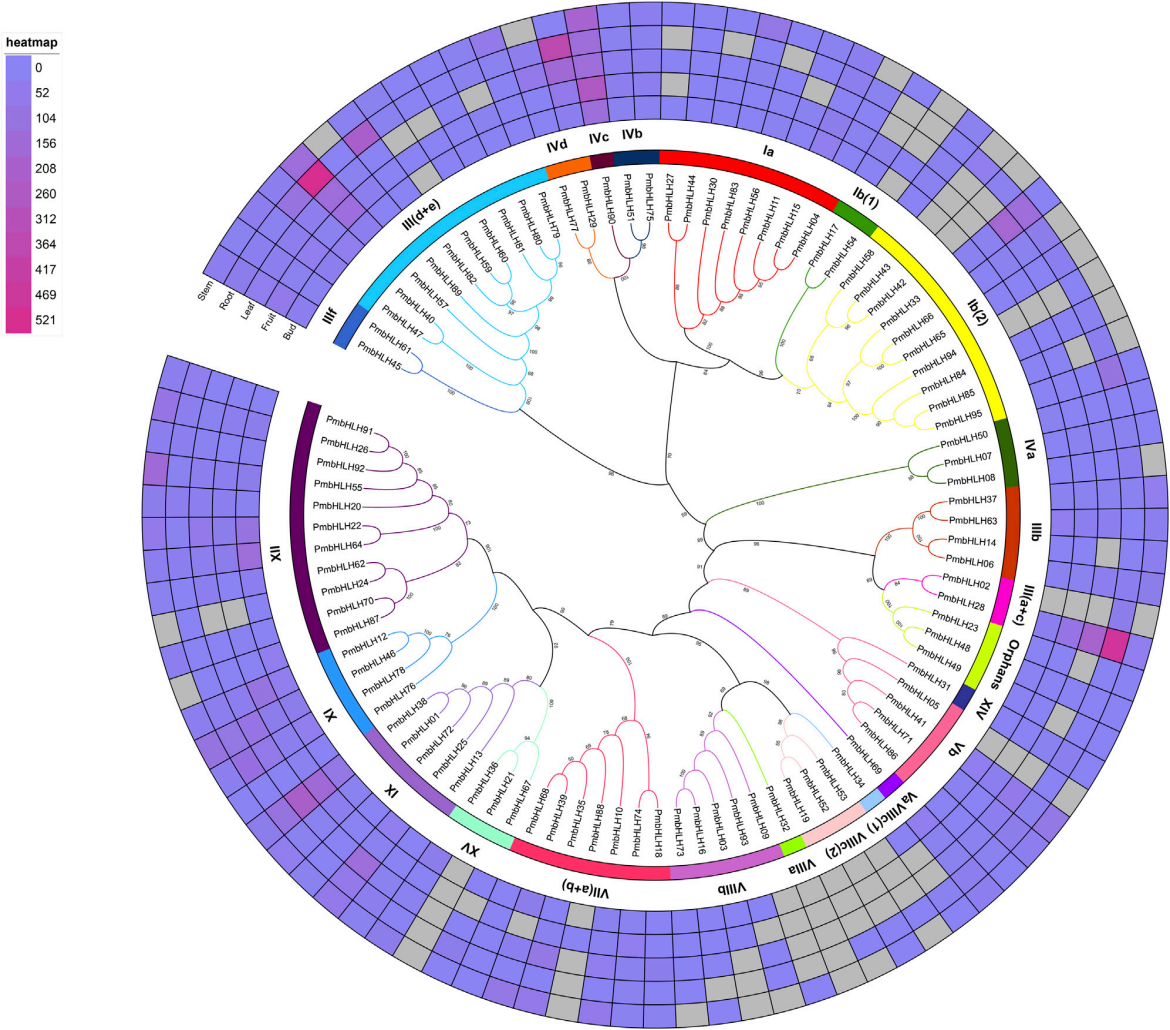
Phylogenetic analysis of PmbHLH proteins in *P. mume*. The RAxML version eight software was applied to draw the Maximum likelihood (ML) tree with 1,000 bootstrap replicates. Lines with roman numerals represent different PmbHLH subfamilies. A heat map of *PmbHLH* genes in five tissues (bud, fruit, leaf, stem, and root) was drew based on a phylogenetic tree. Color scores show the expression of *PmbHLH* genes in five tissues.

To research the evolutionary relationship of PmbHLHs, a phylogenetic tree with ML method was established by full-length amino acid sequences of 95 PmbHLHs (Figure 1). To clearly describe the classification and potential functions of PmbHLHs, we performed another ML phylogenetic tree with 95 PmbHLH proteins and 162 AtbHLHs (Supplementary Figure S1 and Supplementary Table S2). Based on verified AtbHLHs (Pires and Dolan, 2010), 95 PmbHLH proteins were divided into 23 subfamilies and one orphan (Figure 1). XII was the largest subfamily, consisting of 11 members. Five subfamilies [IVc, Va, VIIIa, VIIIc (1), XIV] contained only one bHLH protein. This indicated that PmbHLHs were distributed unevenly in different subfamilies. To date, we know very little about the biological functions of PmbHLHs except for PmICE1 (Cao et al., 2014). However, plenty of bHLH proteins have been confirmed in *Arabidopsis*. Through *Arabidopsis* ortholog analysis, PmbHLHs might play an important role in abiotic stress, hormonal regulation, development, and so on (Supplementary Table S2).

**FIGURE 2:**
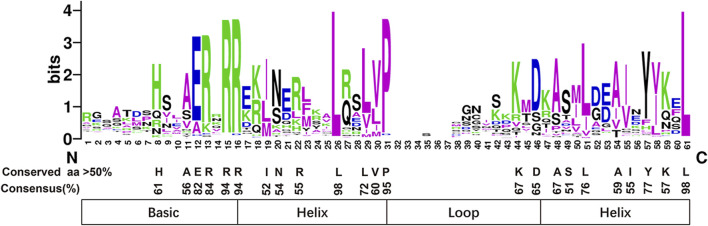
All PmbHLH proteins shown highly conserved in the domain. The overall height of every stack means sequence conservation in the corresponding position. Capital letters represent the conservation of amino acids exceed 50% among 95 PmbHLH domains.

The bHLH domain analysis showed that PmbHLH domains consisted of four conserved regions, including the basic region, two helix regions, and a loop region (Figure 2 and Supplementary Figure S2). Additionally, 23 amino acid residues were conserved (>50% consensus ratio) in their bHLH domains. Among residues, the Arg-15, Arg-16, Leu-26, Pro-31, Leu-61 were highly conserved with a greater than 90% consensus ratio. Among the 23 conserved amino acid residues, the basic region found six conserved residues (His-8, Ala-11, Glu-12, Arg-13, Arg-15, Arg-16), first helix region found seven conserved residues (Ile-19, Asn-20, Arg-22, Leu-26, Leu-29, Val-30, Pro-31), loop region found two conserved residues (Lys-44, Asp-45), second helix region found eight conserved residues (Ala-48, Ser-49, Leu-51, Ala-54, Ile-55, Tyr-57, Lys-59, Leu-61).

The DNA binding activity of target genes was decided by the bHLH domain in the basic region. According to the classification standard in *A. thaliana* (Toledo-Ortiz et al., 2003), PmbHLHs were defined as Non-DNA-binding proteins and DNA-binding proteins (Figure 2 and Table 1). In addition, DNA-binding proteins were divided into E-box-binding proteins (including G-box-binding proteins) and non-E-box binding proteins according to the existence of Glu-12 and Arg-15 (positions were corresponding to positions 13 and 16 in *Arabidopsis*). His/Lys-8, Glu-12. Arg-16 (positions were corresponding to positions 9, 13, and 17 in *Arabidopsis*) are responsible for the binding of the G-box. Based on the conservation of these residues, three PmbHLHs were classified to non-E-box-binding proteins for missing Glu-12/Arg-15 residues and 37 PmbHLHs were classed to E-box-binding proteins. Among 37 E-box-binding proteins, 24 as G-box-binding proteins, while 13 proteins missed the G-box-binding site. Furthermore, 55 of the 95 PmbHLHs were classed in non-DNA-binding proteins due to less than six amino acid residues in the basic region (Table 1 and Supplementary Table S3).

**TABLE 1:** Predicted DNA-binding categories based on the bHLH domain.

Predicted activity	Predicted Motif	Number of PmbHLHs	Number of AtbHLHs (Toledo-Ortiz et al.)
DNA binding	—	—	—
E-box	—	—	—
G-box	bHLH	24 (25.26%)	89 (60.54%)
Non-G-box	bHLH	13 (13.68%)	20 (13.61%)
Non-E-box	bHLH	3 (3.16%)	11 (7.48%)
Total	—	40 (42.11%)	120 (81.63%)
Non-DNA binding	HLH	55 (57.89%)	27 (18.37%)

### Gene Structure and Conserved Motif

**FIGURE 3:**
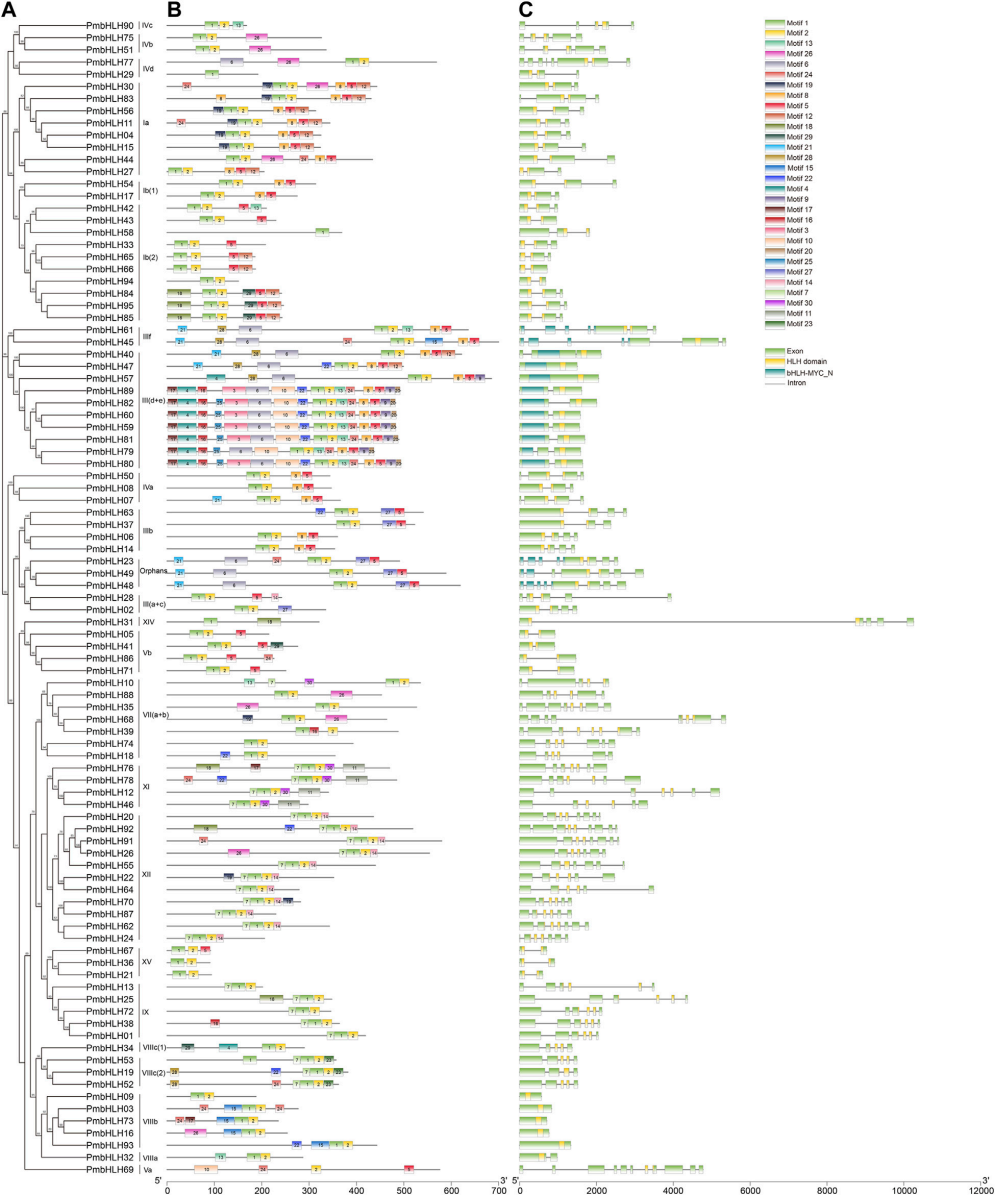
Phylogenetic relationship, motifs analysis, and gene structure in PmbHLH. **(A)** The RAxML version eight software was applied to construct a phylogenetic tree based on PmbHLH proteins. **(B)** Motif composition analysis of PmbHLHs by MEME. Different colorful rectangles with numbers 1–30 represent different motifs. **(C)** Exon-intron structure of PmbHLHs. Grey lines represent introns and green boxes represent exons. Yellow rectangles indicate the position of PmbHLHs conserved domain.

Further to the analysis features of *PmbHLHs*, we investigated intron/exon patterns according to the phylogenetic tree with PmbHLHs sequences (Figure 3A). Analysis of genomic DNA sequences showed that number of introns changing from zero to ten (Figure 3B). Most of them usually had one to eight introns, except *PmbHLH69* and *PmbHLH92*. Different subfamilies had different intron/exon patterns, while the same classes were similar. Seven genes of 95 *PmbHLHs* were intron-less, five of these were in subfamily VIIIb. Seven subfamilies [Ib (1), IIIf, IVb, Vb, VIIIb, IX, XV] had a concentrated number of exons/introns.

We used MEME online tool to predict thirty conserved motifs of 95 PmbHLH proteins (Figure 3C and Supplementary Table S4). The number of PmbHLHs motifs was distinctive, ranging from 1 to 16. Most of PmbHLHs shared three to six motifs. Each PmbHLH protein contained motif one and motif two except for PmbHLH31, PmbHLH58, and PmbHLH69. Different subfamilies had unique motif combinations. For instance, subfamily IVb contained motifs 1, 2, and 26, and subfamily Ib (1) contained motifs 1, 2, 5, and 8. Some motifs were unique and existed in only one subfamily. Subfamily Ⅲ (d + e) contained the most motifs and motif 9, 20, and 25 were specific to it. Motif 11, 23, and 30 were respectively observed in subfamily XI, Ⅷ (2), and XI. These conserved motifs may play special functions. The same subfamily had similar motifs, implying these PmbHLHs might have similar functions. In contrast, the kinds of motifs showed little difference in the same subfamily. For example, PmbHLH07 had motif 21 except common motifs 1, 2, 5, and eight in subfamily IVa.

### Chromosomal Distribution and Synteny Analysis

**FIGURE 4:**
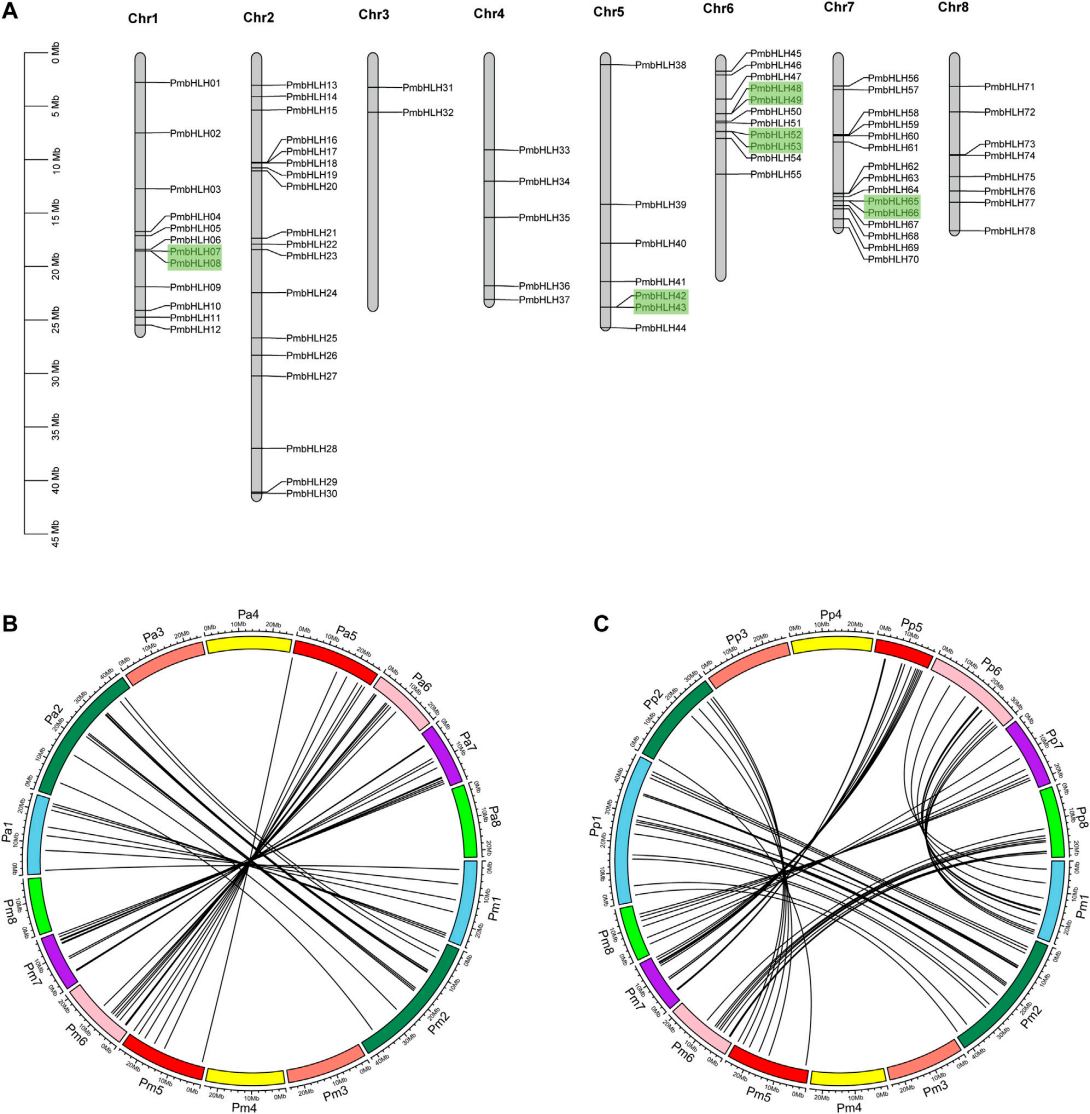
Chromosomal mapping of PmbHLHs and synteny analysis in P. mume (Pm),P. avium (Pa) and P. persica (Pp). **(A)** Chromosomal distribution of *PmbHLH* genes. 78 *PmbHLH* genes were unevenly mapped on eight chromosomes. Tandem duplicated gene pairs are displayed with green blocks. **(B)** Synteny analysis in *P. mume* and *P. avium*. The same color blocks represent the same chromosome label in *P. mume* and *P. avium*. Black lines mean orthologous bHLH in *P. mume* and *P. avium*. **(C)** Synteny analysis in *P. persica* and *P. mume*. The same color blocks represent the same chromosome label in *P. persica* and *P. mume*. Black lines mean orthologous bHLH in *P. persica* and *P. mume*.

According to genome annotation information, 78 *PmbHLH* genes were mapped on eight chromosomes, while 17 *PmbHLH* genes were localized on unassembled genomic scaffolds (Figure 4A and Supplementary Figure S3). The distribution of *PmbHLH* genes on each chromosome was irregular. 18 *PmbHLH* genes (18.95%) were present on chromosome 2, which was the maximum number, whereas only two *PmbHLH* genes (2.11%) were located on chromosome 3. The proportion of *PmbHLH* genes on half of the chromosomes accounted for more than 10% but less than 20%.

Duplication events contained genome duplication, tandem duplication, segmental duplication, and transposon duplication, leading to plant evolution (Qiao et al., 2019). Duplication played an important role in *PmbHLH* gene expansion. Among 95 *PmbHLHs*, nine pairs of *PmbHLH* genes were described as tandem duplication, mapping on chromosome 1, 5, 6, 7, and scaffolds (Figure 4A and Supplementary Figure S3). The largest number of tandem duplications were distributed on Chromosome 6. By contrast, segmental duplication was absent in the *PmbHLH* gene family, which signified segmental duplication was not involved in gene expansion. The selection pressure of gene duplications was estimated by mutation rates of Ka (nonsynonymous) versus Ks (synonymous). We calculated the Ka/Ks of the *PmbHLH* gene family (Supplementary Table S5). Results showed that most of the Ka/Ks values of tandem duplication were <1 and changed from 0.270 to 0.949, implying a purifying selection during *PmbHLH* genes expansion. However, *PmbHLH82*-*PmbHLH83* and *PmbHLH85*-*PmbHLH86* only existed Ka value, which means that two gene pairs evolved by natural selection. The divergence time of *PmbHLHs* tandem duplication ranged from 3.67 to 64.06 Mya.

To further explore the evolutionary mechanism of the *PmbHLH* gene family, we constructed a syntenic map of *P. mume* associated with *P. avium* and *P. persica*. 41 syntenic orthologous gene pairs were distinguished between *P. avium* and *P. mume*, 59 pairs between *P. persica* and *P. mume* (Figure 4B and Figure 4C). This indicates that the *P. avium*, *P. persica,* and *P. mume* had a close relationship. Interestingly, we found that one *PmbHLH* gene only corresponds to one gene in these syntenic orthologous gene pairs. For further evolutionary studies, the divergence time of *bHLH* gene pairs was calculated in three varieties (Supplementary Table S6). Divergence time started 86.11 Mya to 0.67 Mya between *P. persica* and *P. mume*, and 1-2 Mya occurred in most duplicated events. In *P. avium* and *P. mume*, it began 60.81 Mya to 0.14 Mya and 0.5-2 Mya occurred in most of the duplicated events, which may indicate that the speciation time of these orthologous pairs was shorter in the two varieties. Additionally, higher syntenic genes appeared on chromosomes 2, 6, and 7.

### Expression Profile of *PmbHLHs*

Based on the FPKM values from RNA sequencing data, we investigated the expression pattern of *PmbHLHs* among different tissues and different low-temperature treatments. The heatmap of Figure 1 and Supplementary Figure S5 show that the *PmbHLH* gene family presented clear tissue-specific expression. Among 95 *PmbHLHs*, 53 genes were expressed in five tissues (bud, fruit, leaf, stem, and root), implying that these *PmbHLHs* may participate in the development and growth process of tissues. While, *PmbHLH21* and *PmbHLH32* (they belonged to subfamily XV and VIIa, respectively) lacked expression in all detected tissues. We discovered that nine *PmbHLHs* were expressed in only one tissue. They included two genes (*PmbHLH54* and *PmbHLH88*) only in fruit, seven genes (*PmbHLH02*, *PmbHLH05*, *PmbHLH19*, *PmbHLH42*, *PmbHLH43*, *PmbHLH53* and *PmbHLH94*) only in root. This probably indicated that the nine genes have special functions in fruit or root. Moreover, most of remaining *PmbHLHs* were expressed in three or four detected tissues (Supplementary Table S7). According to the phylogenetic tree analysis, *PmbHLHs* from the same subfamily had a similar expression pattern (Figure 1). For instance, IX subfamily (included *PmbHLH01*, *PmbHLH13*, *PmbHLH25*, *PmbHLH38* and *PmbHLH72*) exhibited expression in all detected tissues. Furthermore, Ib (2) subfamily genes (included *PmbHLH42*, *PmbHLH43*, *PmbHLH65*, *PmbHLH66*, *PmbHLH84*, *PmbHLH85*, *PmbHLH94,* and *PmbHLH95*) were highly expressed in root, while *PmbHLH33* and *PmbHLH58* had a high expression level in leaf and stem respectively, implying that the functions of genes in the same family gradually differ in the evolution process.

Previous studies have proved that the cold resistance of apricot mei is stronger than that of true *mume*. Therefore, annual plants of apricot mei ‘Danfenghou’ and true *mume* ‘Beijing Yudie’ were treated with low temperatures. Based on transcriptome data, this study analyzed differences in the expression level of the *PmbHLHs* gene family during these three periods. FPKM value greater than one is an effective expression. 62 genes that were effectively expressed after low-temperature stress were detected. The FPKM value of these genes was plotted as a heat map.

**FIGURE 5:**
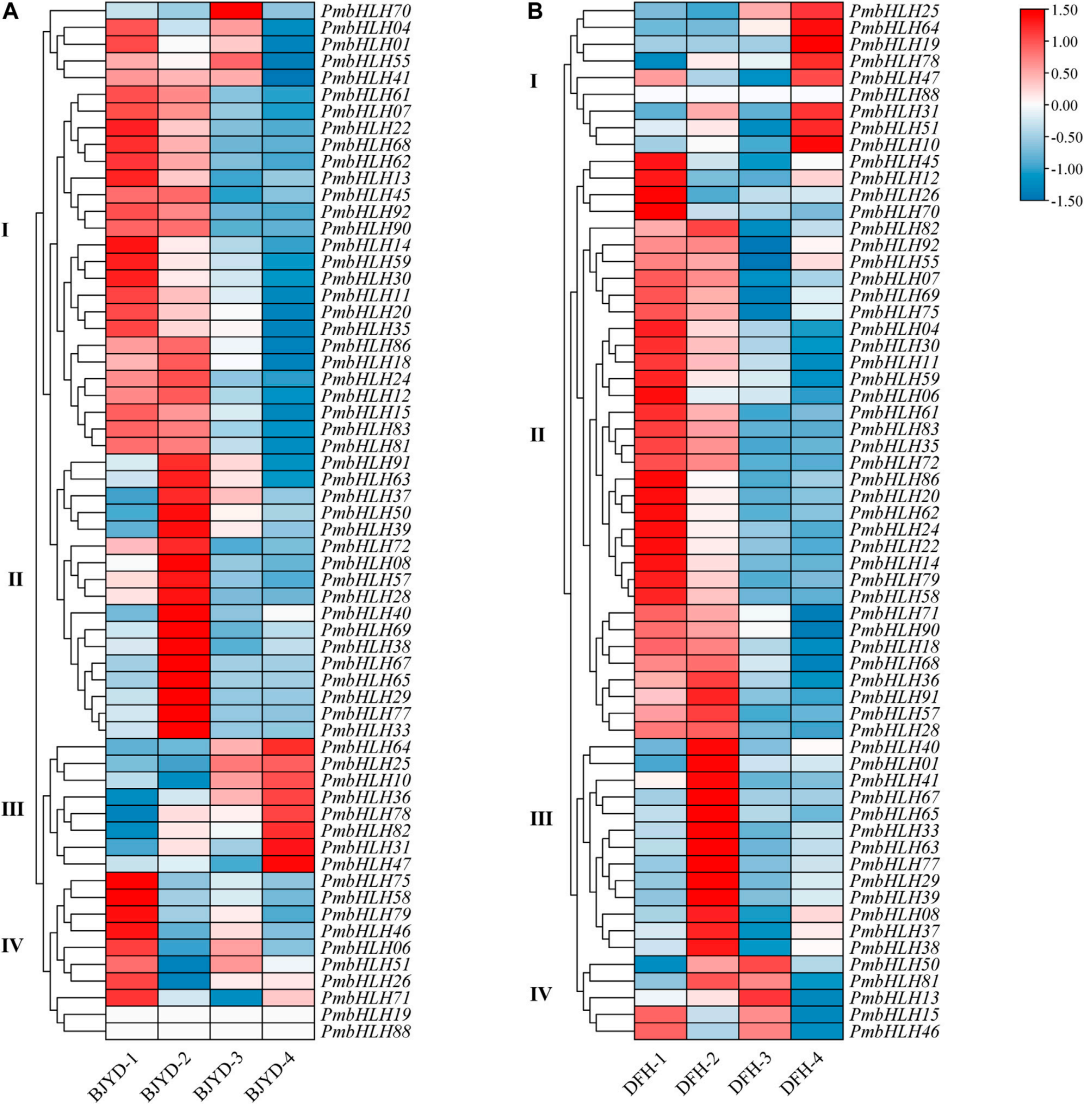
Hierarchical clustering of the expression profile of PmbHLHs in ‘Beijing Yudie’ **(A)** and ‘Danfenghou’ **(B)** under low-temperature treatment. “1” represents stems that were treated at 25°C, “2” represents stems that were treated at 4°C for 8 h, “3” represents stems that were treated at 4°C for 5 days, “4” represents stems that were treated at −5°C for 1 h. Heat maps were generated with FPKM values. Colorful scale means relative expression level and is shown at the top. Red represents high expression and blue represents low expression. Hierarchical clustering groups are displayed by roman numerals.

As shown in Figure 5, according to different expression patterns in different periods, *PmbHLHs* genes in the two varieties were clustered into four groups (Supplementary Table S8). *PmbHLH* genes of the ‘Danfenghou’ group Ⅰ were highly expressed during freezing treatments and had a similar expression pattern to the ‘Beijing Yudie’ group Ⅲ. There are six genes (*PmbHLH64*, *PmbHLH25*, *PmbHLH10*, *PmbHLH78*, *PmbHLH31,* and *PmbHLH47*) in the two varieties with similar expression patterns, which can respond to freezing stress. The expression level of *PmbHLHs* in the ‘Danfenghou’ group Ⅱ and ‘Beijing Yudie’ group Ⅰ gradually decreased with the extension of treatment time. The expression profiles of *PmbHLHs* in the ‘Danfenghou’ group Ⅲ and ‘Beijing Yudie’ group Ⅱ were similar. 14 genes increased expression levels when two varieties were treated at 4°C for 8 h. They might be involved in the perception and transport of cold signals. The expression levels of ‘Danfenghou’ group Ⅱ and ‘Beijing Yudie’ group Ⅰ both increased at 4°C for 5 days, but there were no overlapping genes in the two cultivars. This indicated that the function of *PmbHLH* genes in the two cultivars after prolonged low-temperature acclimation has a difference.

According to FPKM value and fold log2 change value, 10 *PmbHLH* genes were screened out. Compared with control material that has not been treated with low temperature, the transcriptional expression levels of six *PmbHLHs* genes (*PmbHLH25*, *PmbHLH28*, *PmbHLH38*, *PmbHLH40*, *PmbHLH57,* and *PmbHLH78*) increased by 2–5 times at chilling treatment (4°C for 8 h and 5 days), which may be involved in the response of *P. mume* to chilling treatment. The expression levels of *PmbHLH4*, *PmbHLH6*, *PmbHLH26,* and *PmbHLH46* gradually decreased with the extension of 4°C treatment time. Compared with the control, the highest reduction factor reached 5 times. They might negatively regulate downstream low-temperature response genes or proteins to participate in the chilling stress response of *P. mume*. We used transcriptome to further analyze the expression patterns of *PmbHLHs* genes in different varieties. The trend of expression levels with these 10 differential genes showed the same in strong cold-resistant ‘Danfenghou’ and weaker cold-resistant ‘Beijing Yudie’. However, there were differences in multiple variations among different varieties. For example, the expression of the *PmbHLH28* gene in ‘Beijing Yudie’ was 4.8 times higher than that in the control, but only 1.1 times higher in the ‘Danfenghou’ group.

### Expression Analysis of *PmbHLHs* Under Low-Temperature Stress

**FIGURE 6:**
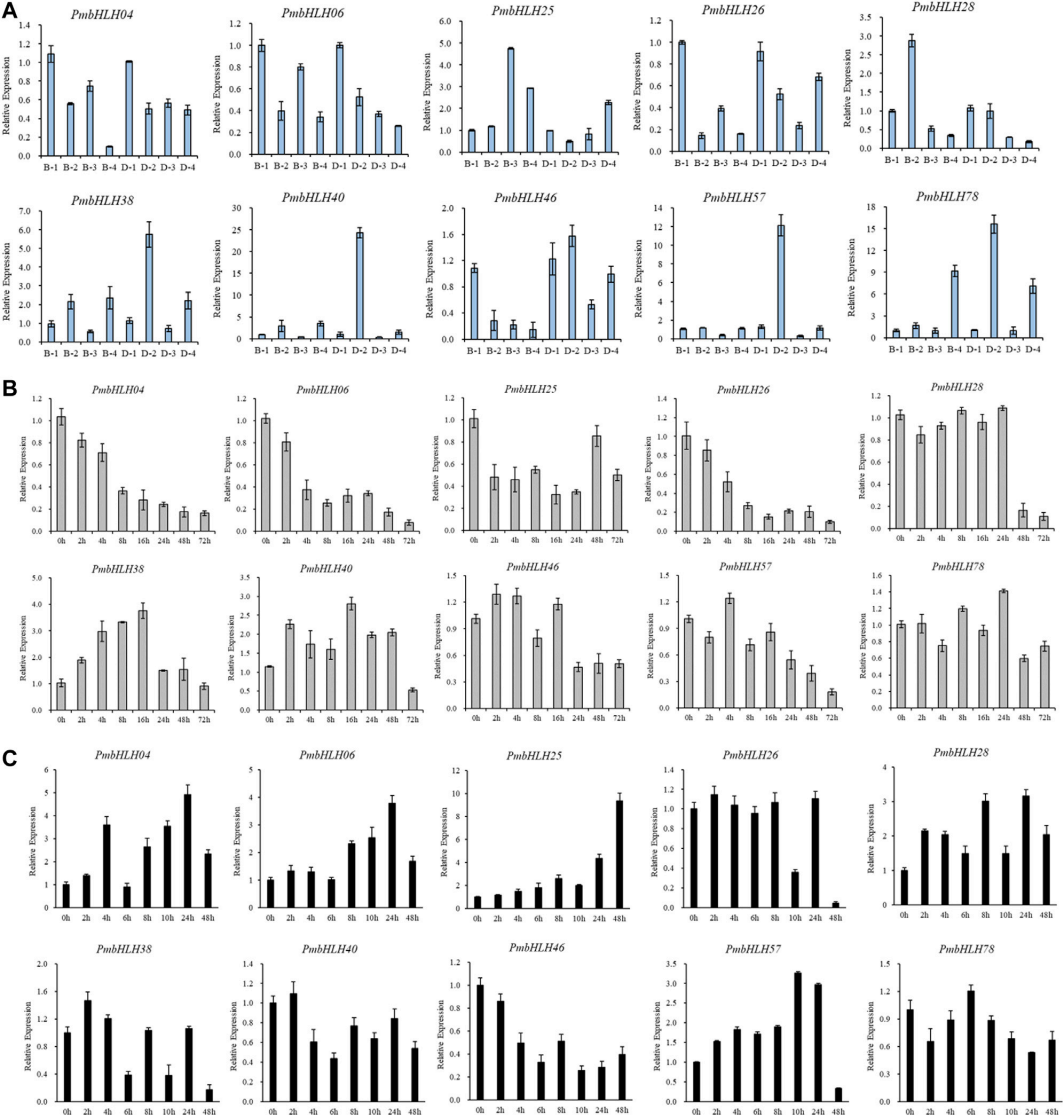
Expression patterns of 10 candidate PmbHLHs in ‘Beijing Yudie’ and ‘Danfenghou’ under low-temperature treatments by qRT-PCR. **(A)** Expression patterns under cold treatment (4°C for 8 h and 5 days) and freezing treatment (−5°C for 1 h). “1” represented stems that were treated at 25°C, “2” represented stems that were treated at 4°C for 8 h, “3” represented stems that were treated at 4°C for 5 days, “4” represented stems that were treated at −5°C for 1 h. **(B)** Expression patterns exposed to cold treatment for different times (0/2/4/8/16/24/48/72 h). **(C)** Expression patterns exposed to freezing treatment for different times (0/2/4/6/8/10/24/48 h). The standard deviation of three independent replicates was represented by Error bars.

To further investigate *PmbHLHs* function in low temperature comprehensively, the 10 genes were detected by qRT-PCR experiments in which the stems of *P. mume* were treated for chilling treatment (4°C for 8 h and 5 days) and freezing treatment (−5°C for 1 h). The expression of *PmbHLH* genes was distinct in two varieties of *P. mume* (Figure 6A). In true *mume* ‘Beijing Yudie’, six genes were up-regulated in varying degrees under low-temperature stress. Remaining genes were down-regulated. Among up-regulated genes, *PmbHLH25*, *PmbHLH40*, *PmbHLH46,* and *PmbHLH57* were highly expressed under 4°C and −5°C treatment. The greatest expression of *PmbHLH25* and *PmbHLH40* was found in 4°C treatments only. In addition, low temperature induced most of the genes to change significantly in apricot mei ‘Danfenghou’. *PmbHLH46* and *PmbHLH25* were up-regulated at 4°C treatment and at −5°C treatment, respectively. *PmbHLH40* and *PmbHLH57* were highly expressed under 4°C and −5°C treatment. In two varieties, four genes (*PmbHLH25*, *PmbHLH38*, *PmbHLH40,* and *PmbHLH78*) could be induced to high expression in true *mume* ‘Beijing Yudie’ and apricot mei ‘Danfenghou’. In addition, the expression of four *PmbHLHs* (*PmbHLH38*, *PmbHLH40*, *PmbHLH57,* and *PmbHLH78*) in apricot mei ‘Danfenghou’ higher than in true *mume* ‘Beijing Yudie’, further showing that apricot mei ‘Danfenghou’ was more resistant than true *mume* ‘Beijing Yudie’.

To explore the regulation process of *PmbHLHs*, we tested the expression pattern of 10 genes by qRT-PCR at chilling treatment (0/2/4/8/16/24/48/72 h) and freezing treatment (0/2/4/6/8/10/24/48 h) for different periods using true *mume* ‘Beijing Yudie’. As shown in Figure 6B, the expression level of *PmbHLH04*, *PmbHLH25*, *PmbHLH26*, *PmbHLH46,* and *PmbHLH57* were reduced with chilling treatment. However, *PmbHLH38* and *PmbHLH40* were induced to high expression and up-regulation expression peaked on treating with 16 h. Under freezing treatment, the expression level of *PmbHLH26*, *PmbHLH40,* and *PmbHLH78* changed slightly. *PmbHLH38* and *PmbHLH40* presented a trend of decreasing. After prolonging the freezing treatment time, *PmbHLH04*, *PmbHLH06*, *PmbHLH25*, *PmbHLH28,* and *PmbHLH57* were induced to high expression. Especially, *PmbHLH25* had the largest expression level increase (approximately 10-fold) at 48 h after being exposed to freezing conditions (Figure 6C). These results imply that *PmbHLH* genes may play a role in resisting low-temperature stress.

### Promoter *Cis*-Acting Elements and Protein Interaction Analysis

The promoter *cis*-elements (1500 bp) were analyzed through PlantCARE software and the predicted regulation mechanisms of *PmbHLHs*. The result showed that *PmbHLH* genes were abundant in abiotic and biotic elements (LTR, MBS, WRE3, WUN-motif, ARE), light-responsive elements (AAAC-motif, ATCT-motif, LAMP-element, Box 4, I-box, and G-box), plant growth and development-related elements (MSA-like, GCN4_motif, and RY-element). In addition, hormone-responsive elements (ABRE, TGACG-motif, G-box, MYC, P-box, and TATC-box) exhibited a wide range of positions in the promoter. This means that *PmbHLHs* may extensively participate in various physiological biochemistry pathways of *P. mume* (Supplementary Figure S4).

**FIGURE 7:**
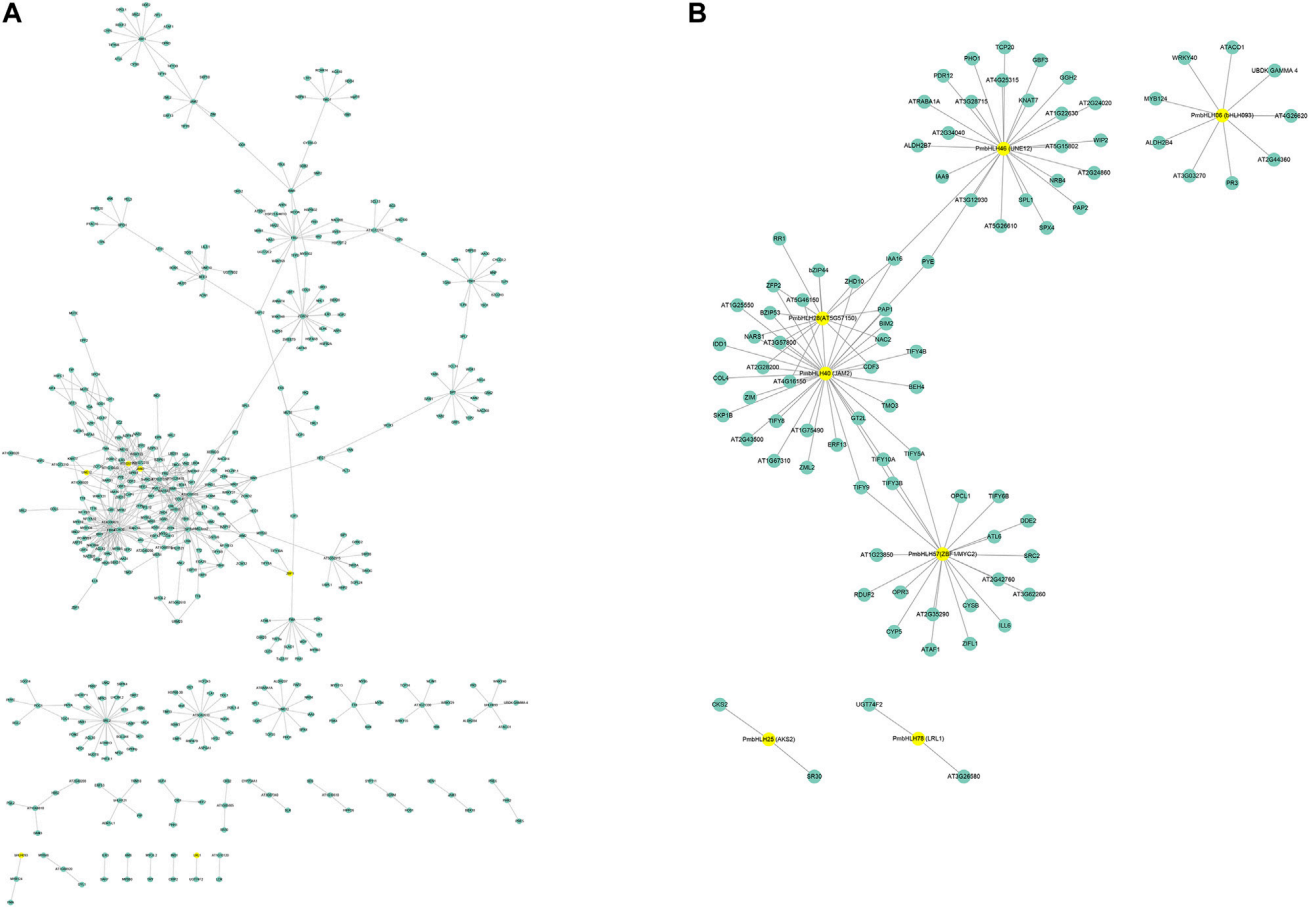
The interaction network for bHLHs in *P. mume* based on orthologs in *Arabidopsis*. **(A)** The whole interaction network for PmbHLHs. **(B)** Protein interaction network of seven candidate PmbHLHs. Yellow circles represent PmbHLHs and blue circles represent other genes.

To further predict the functions of PmbHLHs, we constructed the interaction network using AraNet V2 based on homologous proteins of *Arabidopsis* (Lee et al., 2015). A total of 56 PmbHLH proteins had orthologs in *Arabidopsis* and predicted about 640 interaction protein pairs (Figure 7 and Supplementary Table S9). The interaction network showed that PmbHLHs might interact with MYB, bHLH, NAC, bZIP, HB, WRKY, ERF, and so on, implying PmbHLHs might exert functions by interacting with other genes. The above results predicted that PmbHLHs might be involved in the response of *P. mume* to low temperature. To further research ten PmbHLHs, we constructed and analyzed the interaction network of candidate ten genes. Among the ten PmbHLHs, PmbHLH04, PmbHLH26, and PmbHLH38 were absent in homologous proteins of *Arabidopsis*. The three candidate genes might be novel and potential proteins in response to low temperature. The other seven candidate PmbHLHs formed an interactive network centered on PmbHLH40, which showed high homology to JAM2. Moreover, they might strongly interact with MYB124, TIFY, NAC, WRKY, and IAA to drive function when suffered from low-temperature stress in *P. mume*. Supplementary Figure S6 shows that PmbHLH25 (AKS2) could directly interact with PmbHLH28 (AT5G57150). PmbHLH06 (bHLH93) could directly interact with PmbHLH38 (FBH4). This indicated that these PmbHLH might work by forming dimers under low-temperature stress. Overall, interaction networks could provide a crucial reference for investigating the regulation mechanism of PmbHLHs.

## Discussion

The bHLH transcription factor family plays a positive or negative role in the physiological and biochemical processes of the development of plant trichome, root hair, photomorphogenesis, light signal transmission, and development of plant tissues and organs. In addition, the transcription factor family has contributed to resisting adverse environmental factors in plants, such as drought resistance, salt tolerance, cold tolerance, plant iron deficiency stress, and so on (Toledo-Ortiz et al., 2003). A great many *bHLH* genes have been distinguished in the plant kingdom, including *A. thaliana* (162) (Pires and Dolan, 2010), *Hibiscus hamabo* (162) (Ni et al., 2021), *Helianthus annuus* (183) (Li et al., 2021), *Capsicum annuum* (107) (Liu et al., 2021), *Sorghum bicolor* (174) (Fan et al., 2021), *Osmanthus fragrans* (206) (Li et al., 2020), *Camellia sinensis* (134) (Liu et al., 2021), *Juglans regia* (102) (Zhao et al., 2021), *Pyrus bretschneideri* (197) (Dong et al., 2021). The number of bHLH transcription factors changed widely among various plants. However, the characteristic of bHLH genes remains unknown in *P. mume*. In our research, we used *P. mume* genome to distinguish 95 *PmbHLH* genes. The number of *PmbHLHs* was the same as bHLH in *P. persica*, which further proved that the genetic relationship of *P. persica* and *P. mume* was closer.

*PmbHLHs* were inhomogeneous on eight chromosomes and scaffolds. These results showed that the bHLH gene family existed specific evolution patterns in different varieties. In a previous study, it was suggested that the expansion of bHLH might derive from gene duplication during evolution with a high proportion of segmental duplications and tandem duplications (Cannon et al., 2004; Kavas et al., 2016). In *P. mume*, nine pairs of *PmbHLH* genes were described as tandem duplication (18.95%) and absent in segmental duplications. Tandem duplication possibly promoted *PmbHLHs* expansion. The rate of gene duplication in this study was lower than in other plants, suggesting gene duplication was the secondary formation in *PmbHLH* gene expansion or *PmbHLHs*, and that loss-functions or redundancies may be lost during evolution. This conclusion was similar to the *PmWRKY* genes family (Bao et al., 2019).

The bHLH domain consists of the basic region, two helix regions, and a loop region (Massari and Murre, 2000). DNA binding activity was determined by the basic region of the bHLH domain and the HLH region was essential in homodimer or heterodimer formation (Carretero-Paulet et al., 2010). In the present study, 23 amino acid residues in the PmbHLHs domains were conserved (>50% consensus ratio). Among them, residues Arg-15, Arg-16, Leu-26, Pro-31, Leu-61 (corresponding to Arg-16, Arg-17, Leu-27, Pro-32, Leu-61 in AtbHLHs) showed highly conserved with greater than 90% consensus ratio. Particularly, residues Leu-26 and Leu-61 were more conservative (Figure 2). DNA binding activity in the basic region was decided by greater than five basic amino acid residues (Toledo-Ortiz et al., 2003). According to the classification standard, PmbHLHs were defined as the Non-DNA-binding proteins and DNA-binding proteins (Figure 2). Furthermore, Glu-13 could specifically identify E-box and the position of Glu-13 was stabilized through Arg-16. And the existence of His/Lys-9, Glu-13, and Arg-17 could discern G-box binding motif. Therefore, DNA-binding proteins were divided into non-G-box binding proteins, G-box-binding proteins, and non-E-box binding proteins based on these factors. In addition, Leu-27 and Leu-61 played an important role in protein interaction in the helix region (Carretero-Paulet et al., 2010). 98% PmbHLHs had Leu-26 and Leu-61 (positions were equivalent to positions 27 and 61 in *Arabidopsis*), implying that PmbHLHs possessed dimerization capacity.

Expression profiles of genes could present their functions. Therefore, expression patterns of *PmbHLHs* in five tissues were analyzed. The expression pattern of *PmbHLHs* represented clear tissue-specific expression based on transcriptome data (Figure 1). Among them, *PmbHLH17* and *PmbHLH54* had a high expression level in fruit, suggesting that they might associate with embryonic development. *RGE1* had *PmbHLH17* and *PmbHLH54* homologous proteins in *Arabidopsis*, which could result in the retarded growth of embryos (Kondou et al., 2008). Meanwhile, *PmbHLH27*, *PmbHLH30,* and *PmbHLH44* were expressed in leaf and stem. The homolog *FAMA*, *SPCH,* and *MUTE* could control meristem differentiation during stomatal development (Ohashi-Ito and Bergmann, 2006; Pillitteri et al., 2007; Marcos et al., 2017). In subfamily IIIb, *PmbHLH37* was expressed highly in stem, homolog, and *AtICE* was implicated in cold acclimation response and freezing tolerance (Chinnusamy, 2003). Additionally, *PmbHLH02* were expressed in the root and the expression of *AtFIT* was up-regulated when it suffered from iron deficiency stress in *Arabidopsis* roots (Ling et al., 2002). These results contributed to predicting the functional regions and further understanding the functions of *PmbHLHs*.

Low temperature is an important environmental factor affecting plant yield and distribution. When plants suffer from adversity stress at low temperatures, they could feel low-temperature signals to produce a series of physiological and biochemical reactions and regulate gene expression (Zhu, 2016). Previous reports have demonstrated that the *bHLH* transcription factor had a crucial influence in resisting low-temperature stress (Nakamura et al., 2011). In the present study, we distinguished 10 *PmbHLH* genes that might possess low-temperature resistance based on transcriptome data. By analyzing qRT-PCR, the expression of *PmbHLH* genes was distinct in two varieties of *P. mume*. Among up-regulated *PmbHLHs*, four genes could be induced to express in true *mume* ‘Beijing Yudie’ and apricot mei ‘Danfenghou’. Simultaneously, the expression of four *PmbHLHs* in apricot mei ‘Danfenghou’ was higher than in true *mume* ‘Beijing Yudie’, further showing that apricot mei ‘Danfenghou’ was more resistant than true *mume* ‘Beijing Yudie’. The remaining four genes were down-regulated when they suffer low-temperature stress. To further research the regulation mode of the 10 *PmbHLHs*, fine regulation detection was carried out. The result showed that *PmbHLHs* were more strongly expressed in −5°C treatment than 4°C treatment. In the meantime, the expression of *PmbHLHs* increased with the prolongation of −5°C treatment time, suggesting *PmbHLHs* could respond to deep freezing. Especially *PmbHLH25,* which possibly has great potential in breeding frost resistance. It was noteworthy that *AtAKS2* (ABA-responsive kinase substrates 2) was *PmbHLH25* homologous proteins in *Arabidopsis*. ABA could induce phosphorylation of *AKS* to change stomatal opening or close, then enable plants to adapt to changing environmental conditions (Takahashi et al., 2013). We speculated that *PmbHLH25* might play an essential role in the process of low-temperature signal perception and transduction.

## Conclusion

In our study, we identified 95 *PmbHLHs* from *P. mume* genome and comprehensively analyzed their characterization, including gene identification, gene structure, conserved motifs, DNA binding activity, and chromosomal distribution, protein interaction, synteny analysis, and expression profiling. According to the phylogenetic tree, 95 PmbHLHs were classified into 23 subfamilies. The protein interaction and synteny analysis further expounded the potential functions and evolutionary mechanisms of *PmbHLHs*. Moreover, tissue-specific expression revealed that *PmbHLHs* might widely participate in the development of tissues. Combining with the qRT-PCR date, *PmbHLH04, PmbHLH06, PmbHLH25, PmbHLH28, PmbHLH38, PmbHLH40,* and *PmbHLH57* may play a major role in resisting low-temperature stress. These results provide a molecular basis and valuable insights for further studying the functions of *PmbHLHs* in regulating low temperature in *P. mume*.

## Data Availability

The original contributions presented in the study are included in the article/Supplementary Material, further inquiries can be directed to the corresponding author.
