# Supplementary figures and images for: Genome-Wide Identification and Low-Temperature Expression Analysis of bHLH Genes in Prunus mume
Source: Front Genet. 2021 Oct 1;12:762135. doi: 10.3389/fgene.2021.762135 (PMC8519403; doi:10.3389/fgene.2021.762135)

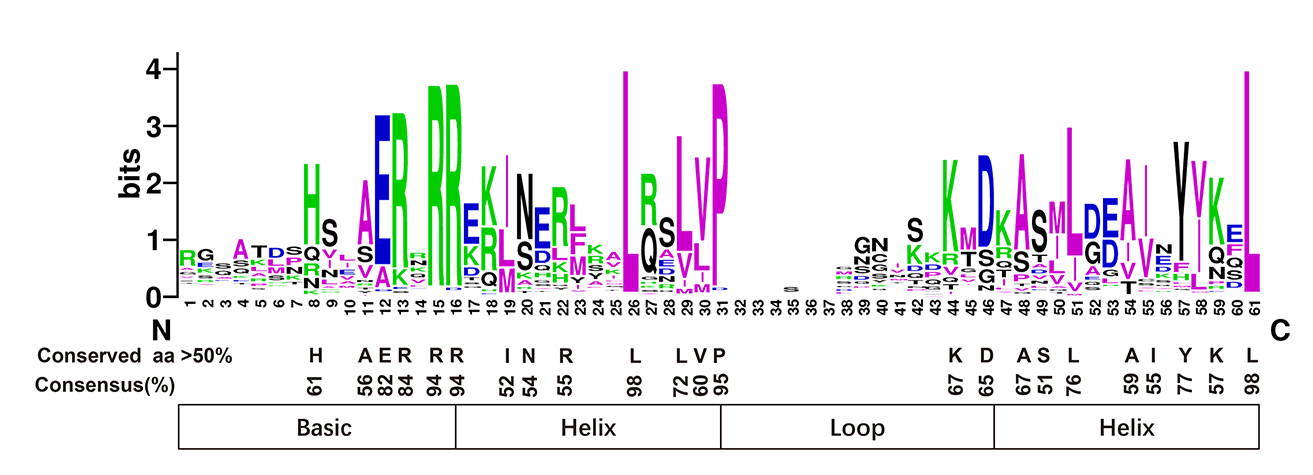

Supplement: Supplementary file 1 [file DataSheet1.ZIP › Figure/Figure2.tif]

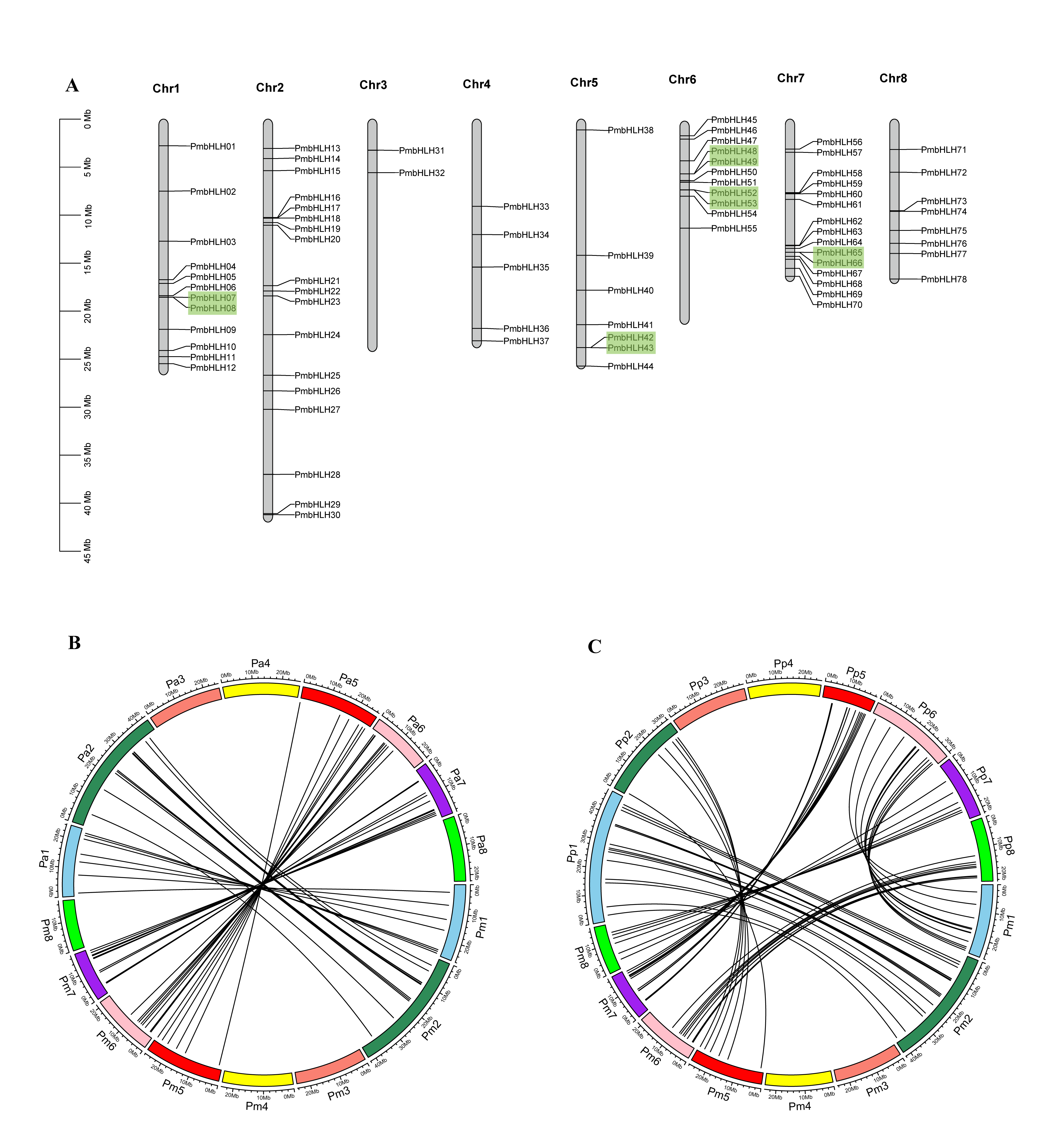

Supplement: Supplementary file 1 [file DataSheet1.ZIP › Figure/Figure4.tif]

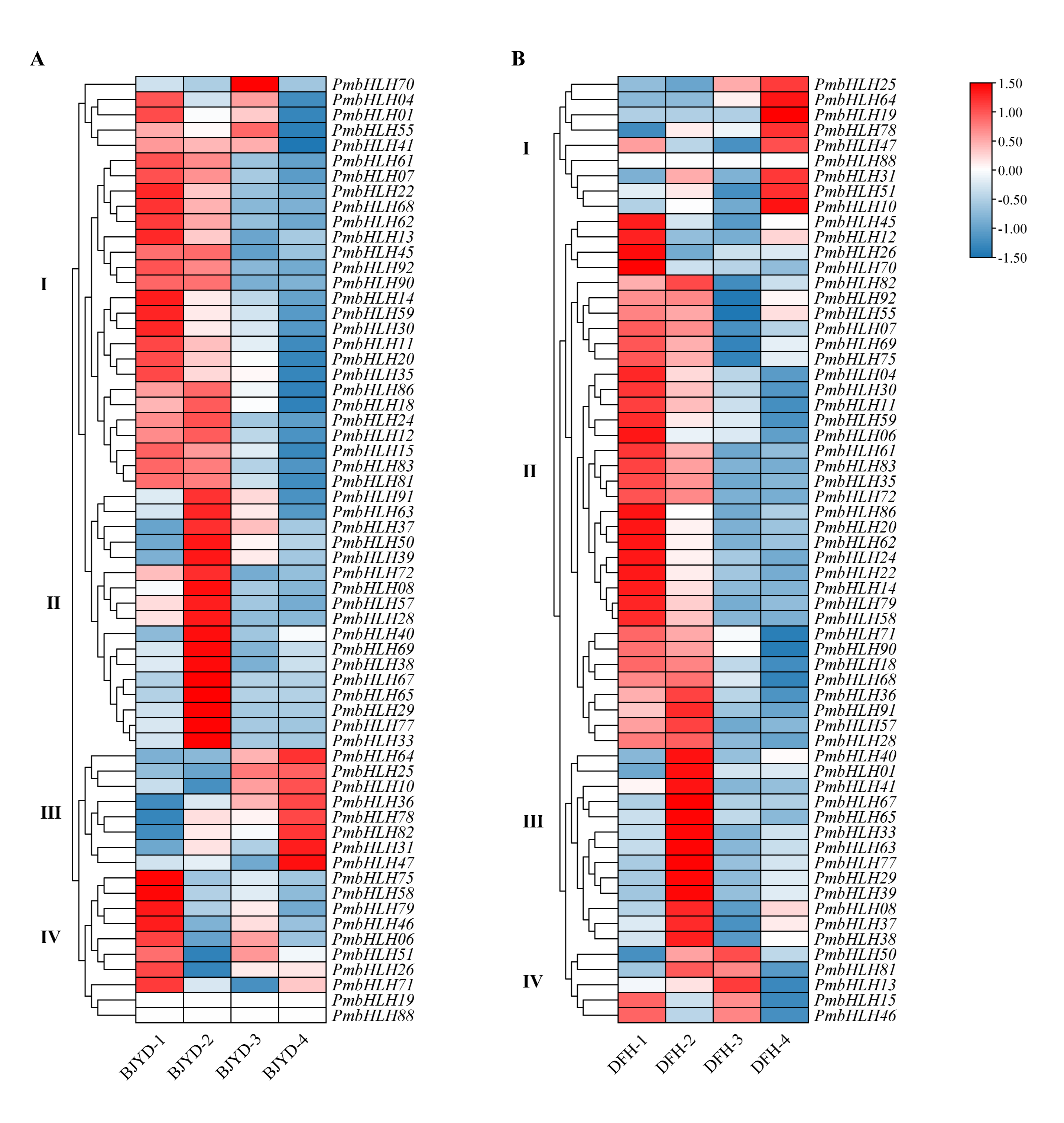

Supplement: Supplementary file 1 [file DataSheet1.ZIP › Figure/Figure5.tif]

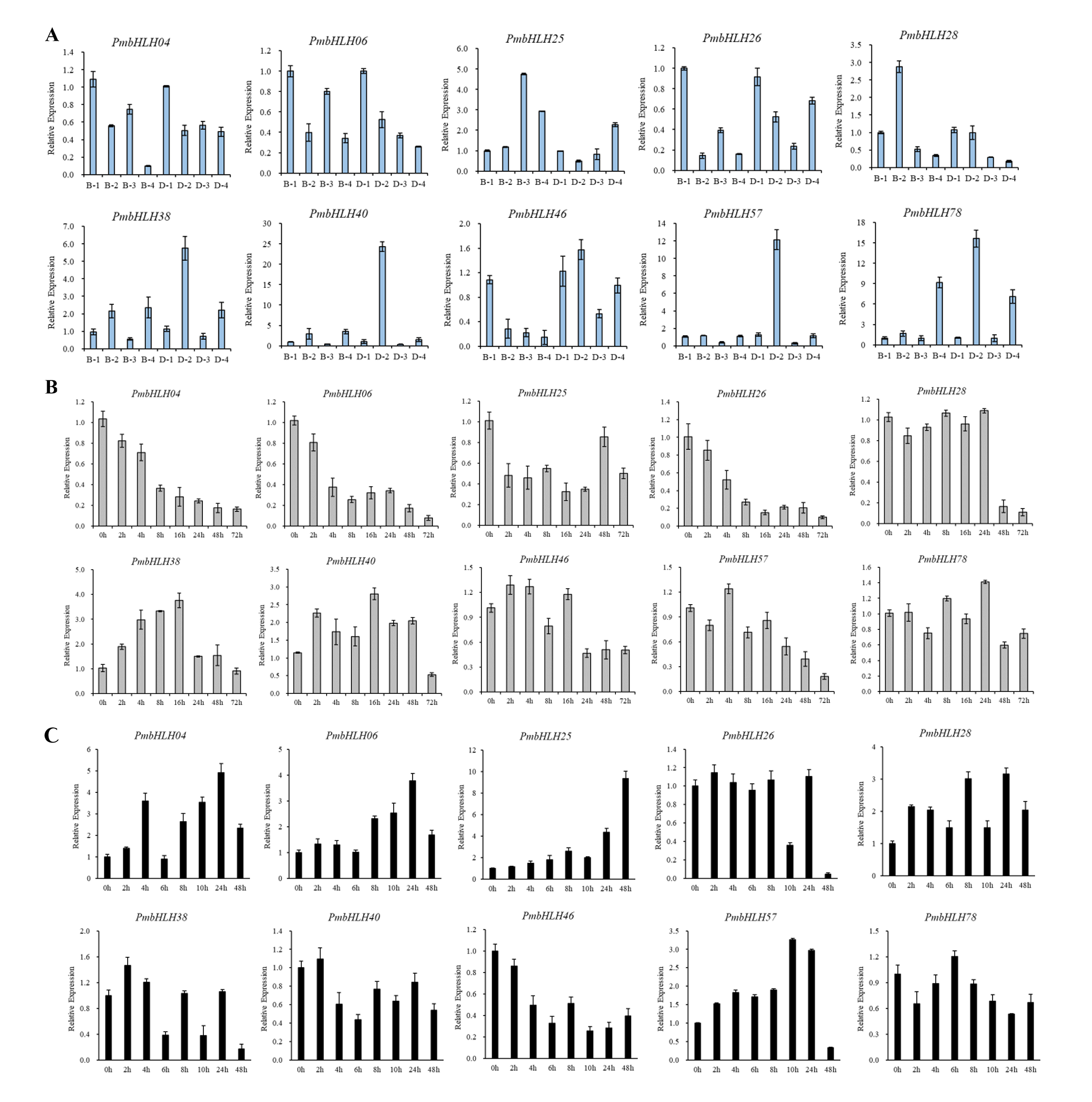

Supplement: Supplementary file 1 [file DataSheet1.ZIP › Figure/Figure6.tif]

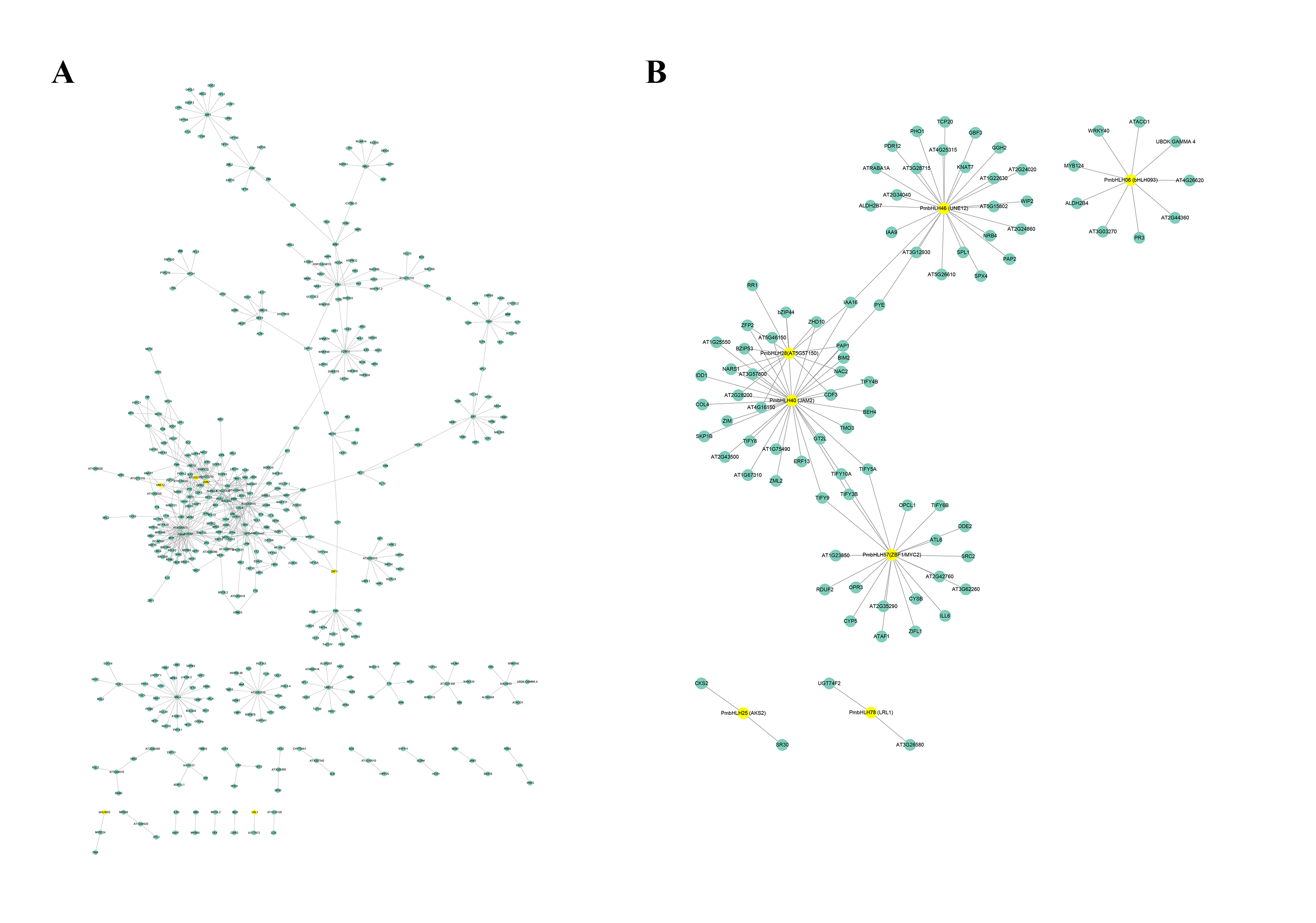

Supplement: Supplementary file 1 [file DataSheet1.ZIP › Figure/Figure7.tif]

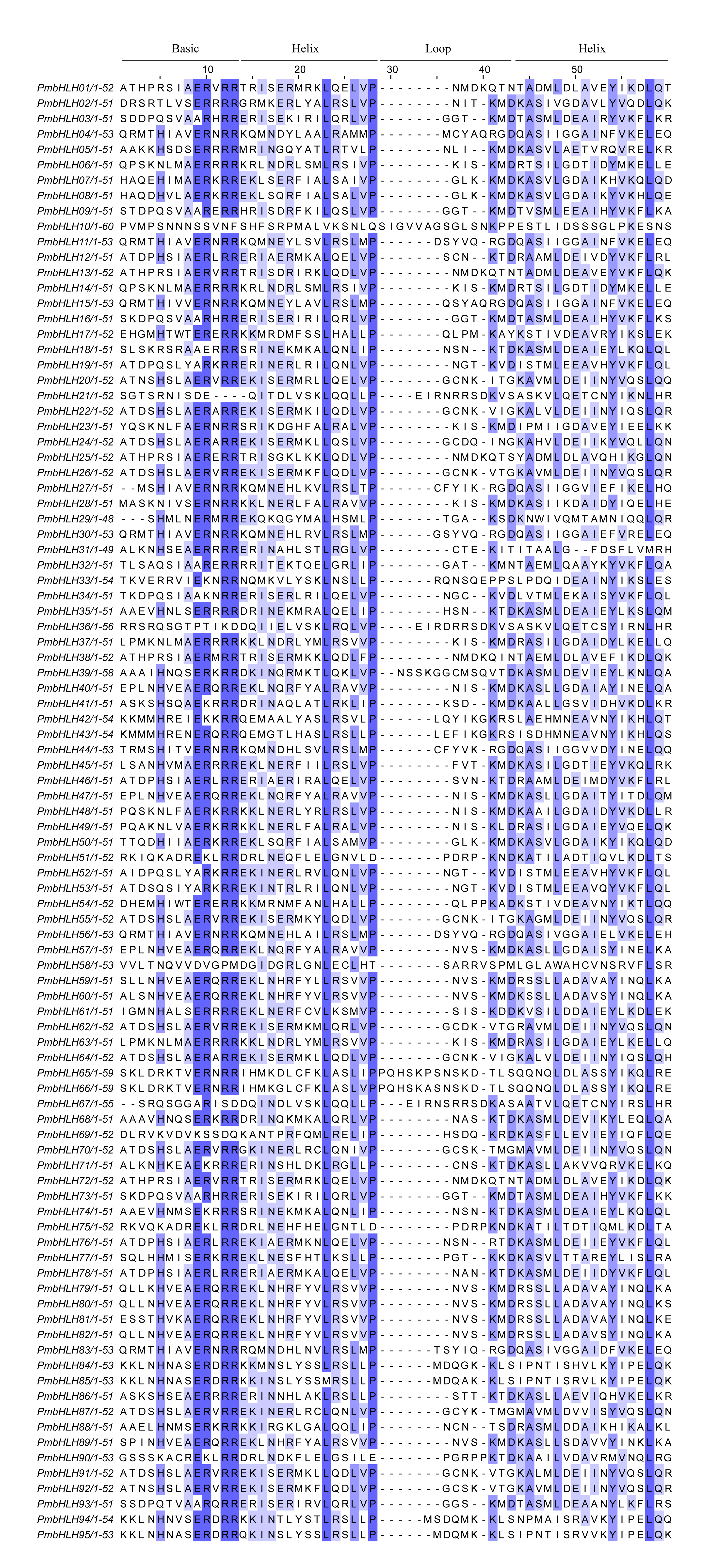

Supplement: Supplementary file 2 [file DataSheet2.ZIP › Supplementary Material/Supplementary Figure 2.tif]

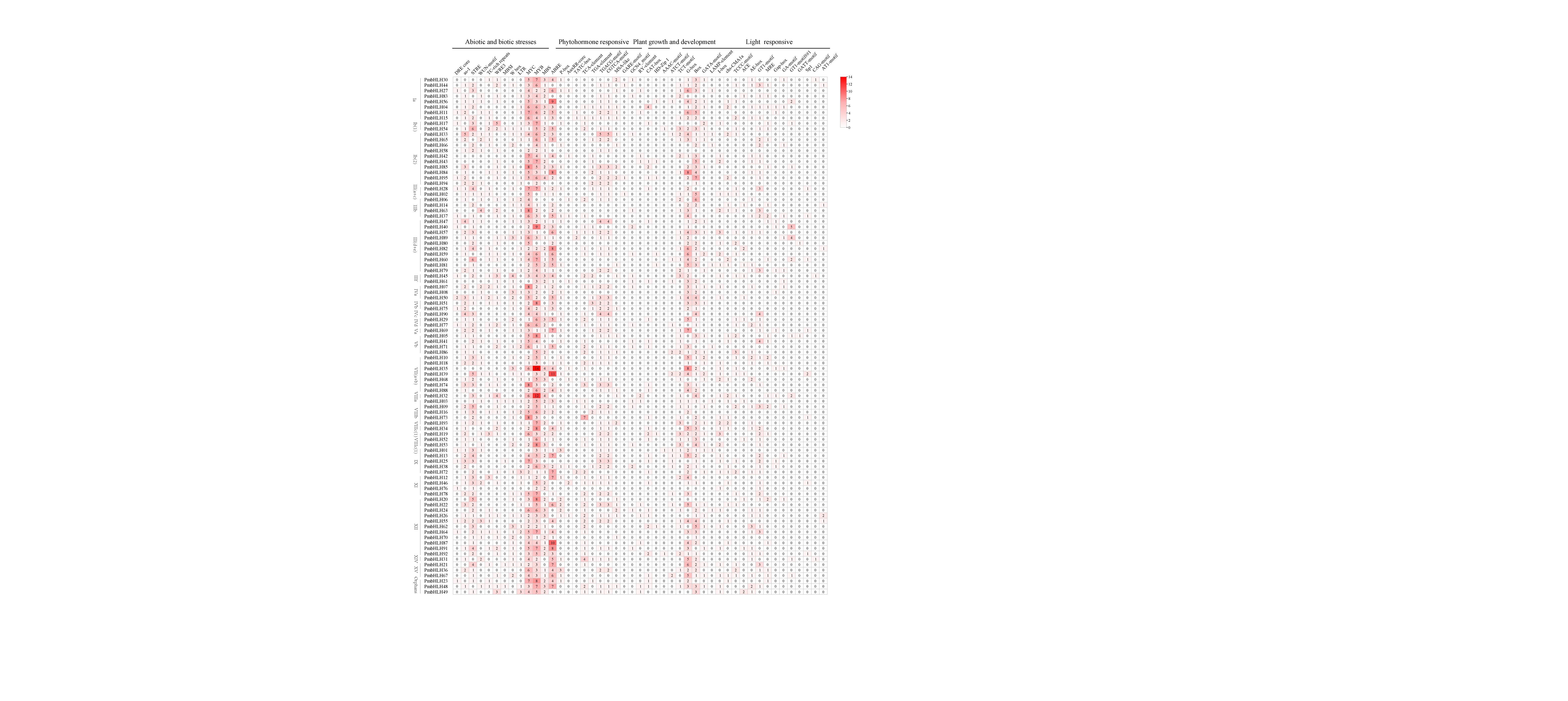

Supplement: Supplementary file 2 [file DataSheet2.ZIP › Supplementary Material/Supplementary Figure 4.tif]

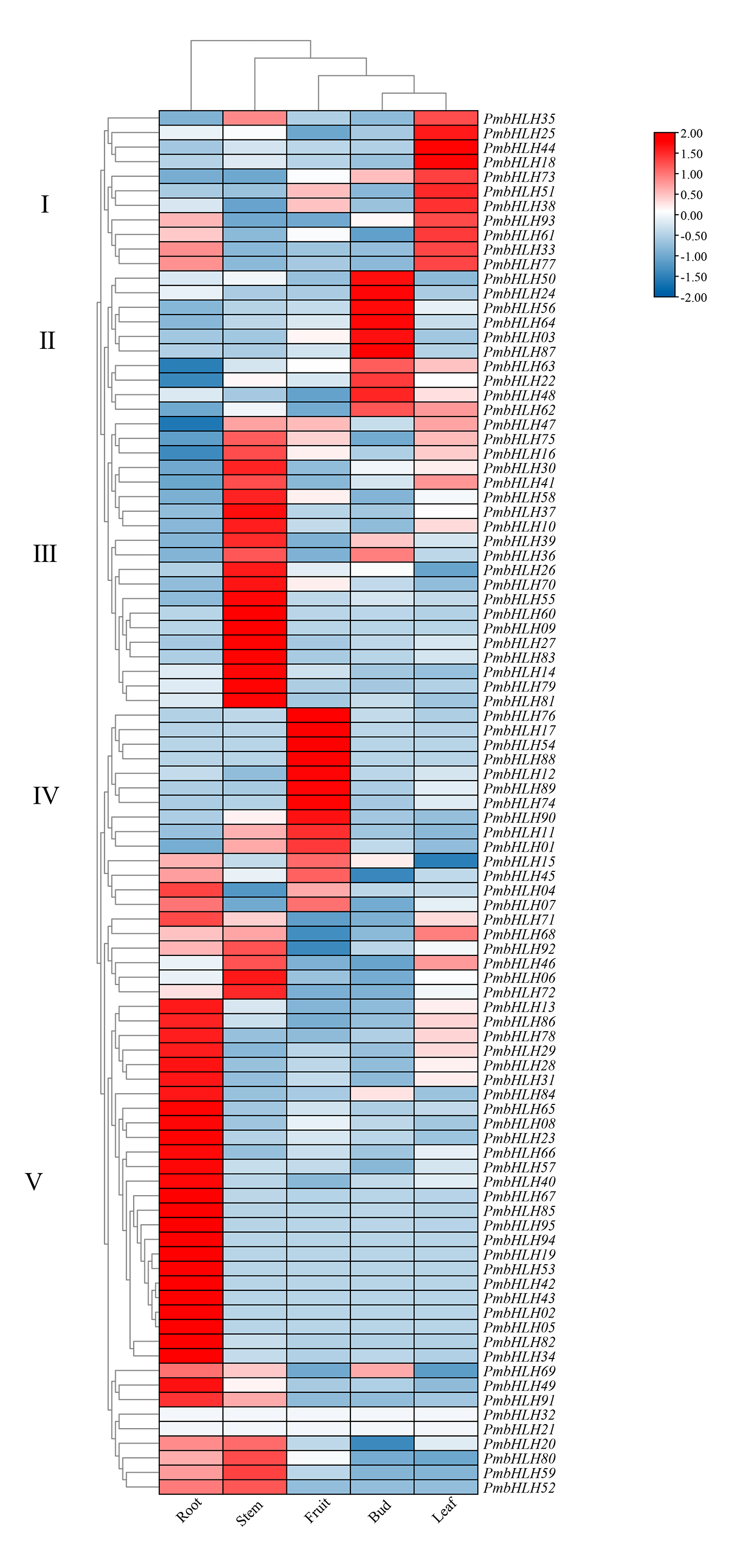

Supplement: Supplementary file 2 [file DataSheet2.ZIP › Supplementary Material/Supplementary Figure 5.tif]

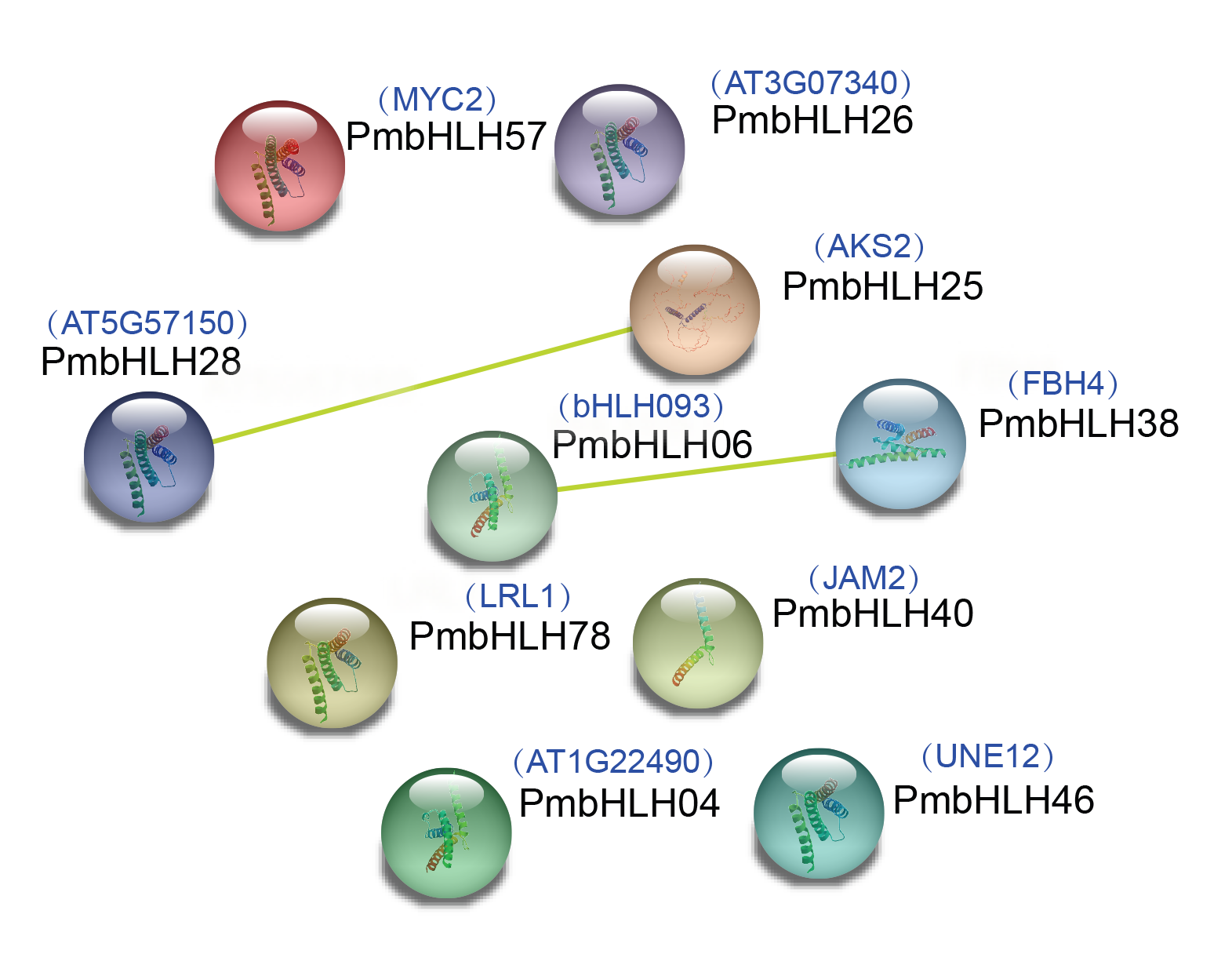

Supplement: Supplementary file 2 [file DataSheet2.ZIP › Supplementary Material/Supplementary Figure 6.tif]
